# Letter from E. R. Hutchins, M. D., Philadelphia, Pa.

**Published:** 1867-12

**Authors:** E. R. Hutchins

**Affiliations:** Philadelphia, Penn.


PHILADELPHIA, PENN., *Nov. 6th, 1867.*

*Editor of Chicago Medical Journal:*—Again, a month has rolled by, and I find myself seated at my desk, endeavoring to add my *mite* to your columns of interest and value. Two cases, of no little worth, among others, have presented themselves to my notice since I last wrote you. The first was that of a miscarriage. I had been engaged by Mrs. P., to attend her in confinement, which she expected would occur in the early part of February, 1868. On the evening of October 7th, about 7 o'clock, I was called to her house, and found her in much pain. From inquiry, I learned she was five months advanced in pregnancy. On the evening previous, she made a misstep on her way from church, and at once felt a pain in her "belly." Never having had a child, she thought not of its being a labor pain. She felt no more of it that night, but on rising in the morning, remarked to her husband that she had "colic." However, she was busy about her house in the forenoon, and in the afternoon, she walked considerably about town, experiencing these "colicy" pains during the whole of the day. About the above time, 7 P.M., she noticed a spot of blood upon some of her undergarments, and at once sent for me. Upon examining her, I found the os dilated to the size of a-half dollar, with the bag of waters protruding. Pains, severe and frequent, were now setting in, and they increased in severity until half-past 2 A.M. of the 8th inst., when I found the os largely dilated and elastic, and I broke the waters. At 3 o'clock, I delivered her of a living child (female.) I was obliged to remove the placenta, being adherent. The child *was washed and dressed, and lived six hours and fifty minutes.* The mother is now quite well. The next case was that of an ovarian cyst, of two years and a-half standing, formed on the person of Mrs. K., aged forty-five. In June, 1867, she presented herself to Prof. Wallace, (through whose kindness I was permitted to see the case,) for treatment. Her abdomen was very much distended, and after preparatory treatment, Dr. Wallace tapped her. At this time, twenty-one and one-half pints of thick gelatinous, brownish fluid escaped. Its specific gravity was 10.2. Eighteen weeks after this operation, Prof. Wallace again tapped her, this time taking eighteen pints of fluid of a like character. On October 20th, she was tapped, and six pints taken. After this tapping, Prof. Wallace, by means of a Davidson's syringe, threw eight ounces of distilled water into the cyst. This was, by the same means, immediately drawn out, together with nearly a pint more of the fluid; then six ounces of ℞. tinct. iodinii ℥j., alcohol ℥xij., aq. distil. ℥vj. Twelve minutes elapsed, from the introduction of the first drop of the iodine, till the last was withdrawn. The pulse was immediately accelerated, but no untoward symptom occurred. The patient was now carefully and comfortably bandaged, and left to enjoy the full anæsthetic effect of the ether which had been administered her. She was, of course, closely watched by Prof. Wallace, and she has had no unpleasant result thus far. Eleven days have now passed since the operation was performed, and there has been no symptom of peritonitis, and there is as yet no sign of the cyst again filling. I have some interesting cases, which I shall from time to time send you.

Yours, &c., E. R. HUTCHINS.

